# A Further Inquiry Concerning Constitutional Irritation, &c.

**Published:** 1837-01

**Authors:** 


THE
BRITISH AND FOREIGN
MEDICAL REVIEW,
FOR JANUARY, 1837.

### PART FIRST.

### Analytical and Critical Reviews.

### Art. I.

1. *A Further Inquiry concerning Constitutional Irritation and the Pathology of the Nervous System.* By Benjamin Travers, f.r.s., Senior Surgeon to St. Thomas's Hospital, &c.—*London,* 1835. (Part II. *On the Pathology of the Nervous System.*)

2. *Outlines of Human Pathology.* By Herbert Mayo, f.r.s., Professor of Anatomy and Physiology, and Pathological Anatomy, in King's College, London; Surgeon to the Middlesex Hospital.—*London,* 1836. 8vo. pp. 595. (Chaps. V. VI. and VII. *Of the Nerves, the Spinal Cord, and the Enkephalon.*)

3. *An Essay on the Laryngismus Stridulus, &c.; to which are appended, Illustrations of the General Principles of the Pathology of Nerves, and of the Functions and Diseases of the Par Vagum and its principal Branches.* By Hugh Ley, m.d., Member of the Royal College of Physicians of London, Physician to the Westminster General Lying-in Hospital, &c.—*London,* 1836. 8vo. pp. 480. (Appendix: *On the Pathology of the Nerves.*)

4. *Pathological and Practical Researches on the Diseases of the Brain and the Spinal Cord.* By John Abercrombie, m.d., Fellow of the Royal College of Physicians of Edinburgh, &c., and First Physician to his Majesty in Scotland. *Third Edition, enlarged.—Edinburgh,* 1835. 8vo.

5. *Lectures on the Nervous System and its Diseases.* By Marshall Hall, m.d. f.r.s. l. and e.—*London,* 1836. 8vo. pp. 171.

6. *Medico-Chirurgical Transactions; published by the Royal Medical and Chirurgical Society of London.* Vol. XIX.—*London,* 1835. (1. *On Serous Effusions from the Membranes and into the Ventricles of the Brain, and its Connexion with Apoplexy and other Diseases of the Brain;* 2. *On Hypertrophy and Atrophy of the Brain;* 3. *On the Cure of Ramollissement of the Brain.* By J. Sims, m.d. &c.)

7. *Isis Revelata: an Inquiry into the Origin, Progress, and present State of Animal Magnetism.* By J. C. Colquhoun, Esq., Advocate, f.r.s. e.—*Edinburgh,* 1836. Two vols. 8vo. pp. 395, 416.

It was naturally to be expected that the important discoveries which have been made of late years in the physiology of the nervous system,—the experimental demonstration of the principle that its different parts, nay, even in many cases the different component fibrils of a single nerve, or parts of a single fibril, possess perfectly different endowments,—and the determination, in many instances, of the specific functions of these parts,—should give a new stimulus to the corresponding department of pathology, and occasion various attempts to give precision and accuracy to that important branch of medical science: and the works, of which the titles are prefixed, appear to us the most important of those which have been composed with this view, or had this tendency, of late years in our own country.

In endeavouring to give our readers some account of these works, and afterwards of one or two others, we shall arrange the most original portions of the information which they contain, as well as we can, according to their bearing on the elucidation of the following general questions:

1. How far is the nervous system concerned in producing or maintaining the diseased actions of the organs of *organic* life, or those diseased states in which the organic functions appear chiefly in fault?

2. What is the exact state of our knowledge as to the pathology of the Nerves; i. e. as to those diseased states of the *animal* functions which originate and are chiefly seated in the nerves?

3. What is the state of our knowledge as to the pathology, similarly defined, of the spinal cord and medulla oblongata?

4. What is the state of our knowledge as to the pathology of the brain or parts of the nervous system superior to the medulla oblongata?

I. The first of these enquiries involves, of course, the fundamental question in physiology, whether the nervous system is directly and essentially concerned in maintaining the *organic* life of animals, (i. e. those functions of animals which go on without the intervention of sensation, or of any other mental act;) or whether the organic life is truly *dependent* on the nervous system only in so far as it is dependent, in the adult state, on sensation and instinctive action, and therefore on a portion of those strictly animal functions which all admit to be, in the more perfect animals, connected with the nervous system exclusively.

Mr. Travers is the only one of the authors before us who gives a decided opinion on this fundamental point; and he thinks it necessary to make a sort of apology for his adherence to the now somewhat unfashionable doctrine of the necessary intervention of an influence derived from the nervous matter in every act even of organic life. We cannot observe, however, that he has stated any new fact in favour of the opinion which he holds in common with Cullen and others of the last age on the subject; and his statement of the reasons which induce many—we may say most—of the physiologists of the present day to reject that doctrine, appears to us decidedly imperfect and inaccurate.

Mr. Travers, it will be observed, not only maintains that some change in nervous matter is an essential element in every vital action, but adheres to the old non-specific doctrine of a nervous influence or energy, seated in the brain and ganglia, conducted along the nerves, and vivifying every part to which it is supplied. "The brain and ganglia," he says, "are organs furnishing a material impalpable to our observation, of which the fibrous structure we call nerves are conductors." Again, "If the brain is a secreting organ, its subservience to the circulation must be that of all such organs; its working or failing, the healthy or vitiated condition of its product, must depend on the sufficiency and regularity of its nourishment by the blood, as much as that of the liver; and the action of the heart as a muscle can only be maintained by a supply of that nervous element which contributes its sensitive and motive power derived directly or indirectly from the same source." "The actions of the respiratory, circulatory, secretory, and excretory organs, the temperature, the tone of the mind and of the muscles, are *all immediately depending* on the condition of the nervous system." (P. 258-9, 260.) Again, in his final summary of conclusions, he says, "The impregnation of the blood with the nervous principle I believe to be as essential to the maintenance of life as its impregnation with the oxygen of the atmosphere, whether we regard nourishment, the production and maintenance of animal temperature, or the diseases incidental to its morbid conditions." "The mechanical contrivance of the blood's distribution is subordinate to its due preparation to nourish and excite the nervous organ; and *through this indispensable medium* all the organs of which the body is composed." He adds, somewhat dogmatically, "I do not separate, in my view of the subject, the motive power of muscles from the nervous agent. It solves no difficulty, but rather creates one, to imagine an independent motion, by whatever term designated, resident in any structure. The reaction of the nervo-muscular solid upon the innervated blood, or that which is duly prepared by the nervous agent, supersedes the *unphilosophical hypothesis* of an independent motivity of tissue." (P. 424-5.)

Now, with all due respect for Mr. Travers, we think we shall command the assent of most of our readers when we assert that Haller's doctrine of irritability, which he here attacks, is both more philosophical and more distinctly intelligible than that modification of the doctrine of nervous influence or energy which he would substitute for it. That muscles in the living body, duly supplied with arterial blood, contract in a peculiar manner on the application of stimuli, is a *fact*. When we attribute to them the vital property of irritability, we do no more than assert that fact; and when we lay down the laws regulating that property, we do no more than state the different conditions and modifications under which that general fact has been observed. That one of the conditions essential to the exercise of this property of irritability is an influx of an imperceptible nervous influence from the brain or the ganglia, or of "innervated blood," or any preliminary change in the nervous system, is an *opinion* or hypothesis as to the cause of that property of irritability. The hypothesis may be true or may be false; but, until it is *proved* to be true, we maintain that it is strictly philosophical, and in accordance with the practice of philosophers in all other sciences, to speak of the property of irritability as belonging to that texture which is *seen* to exhibit it; to regard its existence then as the ultimate fact hitherto *known* on the subject, and then to consider carefully any attempt that may be made to resolve it into a more general fact, or explain it by means of ascertained properties of any other texture. In the mean time, as it appears to us equally easy to conceive the power of contraction to reside in a living muscle, as to be given it by its nerve, we may reverse Mr. Travers's position, and say that it solves no difficulty, but rather creates one, to suppose that the contractile power of muscles is derived from nerves.

According to this, which we maintain to be the only philosophical mode of procedure, it is incumbent on those who maintain that irritability is the "reaction of the nervo-muscular solid on the innervated blood," to *prove* that some such intervention of the nervous system is essential to its manifestation; and for such proof we look in vain, either in the work of Mr. Travers or of other more strictly physiological writers. That the contractions of muscles in obedience to the will or to the sensations of the animal are effected through certain of their nerves, is indeed certain; but we speak here of those contractions (in whatever muscles they may be seen,) which are neither excited by volition, nor preceded by sensation, nor attended with consciousness, but which nevertheless perfectly exemplify the property of irritability, and, in the case of the strictly involuntary muscles, are the sole mode of its exercise in the healthy state. Mr. Travers alleges that "it is no real refutation of his statement" (ascribing them to the 'nervous agent,') "to cite the examples of a continued or a renewed action of the heart detached from the body, by aid of external agents, the nature of which has some affinity to that of the nervous agent. If it were independent of the nervous supply, why is there so brief a term to the duration of this action?" But the question here put is easily answered; and Mr. Travers must surely be aware that the fact here stated is not that on which the opponents of the doctrine of a nervous influence supplying irritability to the muscles take their stand. When the heart or any other muscle is detached from the body, it loses at once its *real* perceptible supply of arterial blood, and its *supposed* imperceptible supply of nervous influence. Here an *experimentum crucis* presents itself. If the term to the duration of the action is brief only because the influence, of which the *nerves* are conductors, is cut off, it should be equally brief, although the muscle remain in the living body, if all its nerves are cut, or the sources of the nervous supply removed. But the reverse is the fact. The heart continues its actions unchanged, although its nerves be cut, although the ganglia whence these nerves come be destroyed; and, if artificial respiration be employed as a substitute for the natural, or if the condition of the animal be such that natural respiration is not essential to the degree of vitality which it enjoys, it continues its actions notwithstanding that both the brain and spinal cord are cut out of the body or *gradually* destroyed, for many hours together, and until the oxygenation of the blood supplying it has obviously become very much impaired. These points have been ascertained by Dr. W. Philip, Dr. M. Hall, and others. What is still more decisive, a muscle which has been stimulated by galvanism until its irritability has appeared to be exhausted, gradually recovers that irritability, and becomes as susceptible of the stimulus as previously, notwithstanding that all its nerves have been divided, if it be merely supplied with arterial blood and its organization maintained.* It is not, therefore, the *brief duration* of the irritability of a muscle cut out of the body, but it is the *indefinite duration* of the irritability of a muscle of which all the nerves have been cut, *so long as it lies in the living body, and receives its usual supply of arterial blood,* which, in our judgment, forbids us to entertain an idea of that irritability being supplied to it from the brain and ganglia, and conducted through its nerves. Mr. Travers indeed may say that, in this case, it has a supply of "innervated blood;" but what proof has he of this? And if he has no proof, who is it that proposes an "unphilosophical hypothesis?"

But, it may be said, injuries confined to the nervous system suddenly affect, or even irretrievably destroy, the functions of the organic life; and this implies an immediate dependence of these functions on the nervous system. The fact is true, and is of great pathological importance; but the physiological inference is more than it warrants. Here, again, the most important physiological fact is the effect of the section of nerves. If the *sudden* suppression of the heart's action, effected, as in Legallois's experiments, by crushing the spinal cord, or in Dr. Wilson Philip's, by crushing the brain, had depended merely on stopping a nervous influence passing from these organs to the heart, it never could have happened that the heart's actions, under the artificial respiration, would go on with unaltered force for hours together, after the brain has been removed from the body, as in the experiments of Fontana, Cruikshank, Brodie, and many others; or after both brain and spinal cord have been removed from the body, or gradually but completely destroyed, as in the various experiments of Flourens, Wilson Philip, and Marshall Hall. On the supposition of the nervous energy being essential to vital action, the one mode of

* See Experiments by Dr. J. Reid, in the Transactions of the British Association, vol. iii.

injury should have been as quickly fatal to the heart's action as the other. These facts clearly prove that, although a part of the nervous system be not essential to an act of organic life, yet a violent injury of that part may be fatal to that portion of the organic life; and therefore that we cannot trust to the effect of violent injuries, as indicating what is the real state of dependence of parts on each other in the natural and healthy state of the living body.

Again, it may be said that, although no influence or energy is transmitted through the nerves to the muscles, to enable them to contract, yet the nerves of muscles themselves (apart both from brain and ganglia,) are necessary intermediate links in the process by which these muscles are excited; that all stimuli act first upon them, and through them on the muscular fibres. This is a very different doctrine from that of the secretion of the nervous power in the brain or in the ganglia, and its transmission through the nerves to vivify the muscles; and it is maintained by higher authority. This was the doctrine as to the connexion of nerves with involuntary muscular motion, maintained in the last age by Whytt, and in the present by Cuvier and Tiedemann. We can give no *proof* that it is a false doctrine, but we think it quite clear that it is an hypothetical doctrine; and, until some clear evidence of the intervention of a nervous change preliminary to the muscular contraction is adduced, we hold it to be perfectly fair and strictly philosophical to decline saying more in regard to it than "affirmantibus incumbit probatio."

We may, however, further remind the advocates even of this form of the doctrine of the dependence of irritability on nerves, of two known facts which are not easily reconciled with it. If all stimuli acted on muscles only by first impressing the nerves which enter them, we might expect to find, 1, that all stimuli which excite muscles when applied to their own fibres would excite them also if applied to the nerve, or to certain of the nerves entering these muscles; and, 2, that all muscles would have nerves, the stimulation of which, in one way or other, would be effectual in exciting the muscles: but neither of these expectations is fulfilled by observation of nature. Although muscular fibres are excited both by mechanical and chemical stimuli applied to themselves, it is the former class of stimuli only, according to the observation of Dr. Wilson Philip, which are effectual even on the muscles of voluntary motion, if applied *exclusively* to their nerves.* And, although the heart is at least as irritable to stimuli applied to itself as any other muscle, we need hardly say that the very general result of the experiments of physiologists is, the absolute inefficiency on the heart's actions of stimuli confined to the nerves, or the nervous ganglia from which it is supplied.

If, therefore, we confine our inferences on this subject to the

* On Sleep and Death.

expression of facts which are susceptible of demonstration, (which, we humbly apprehend, is the only sure way of avoiding unphilosophical hypotheses,) we shall assert that muscles are irritable, and that the irritability of muscles is capable of being excited in many instances, and variously affected in other, perhaps in all instances, in the living body, by causes acting on certain parts of the nervous system; but we shall reject the doctrine of a nervous matter secreted in the brain or in the ganglia, and passing down the nerves, to give this irritability to the muscles; and we shall suspend our belief as to the alleged necessity of any nervous change uniformly preceding muscular contraction, until we have more decisive facts to determine that question.

In regard to the other great division of the functions of organic life, the vital actions of nutrition, secretion, and absorption, although the question as to their dependence on a nervous influence does not stand exactly on the same footing, yet, if we follow the same rule of extending our inferences from the facts known no further than these facts necessarily warrant, we shall find it equally incumbent on us to set aside that doctrine of a nervous influence essential to nutrition or secretion, transmitted through the nerves to the part where these changes take place.

It is true that these functions are more powerfully affected, in various instances, by section of nerves, than the function of involuntary motion is; but, when we attempt to infer that they *depend* on an influence communicated along the nerves, we are immediately met by the following objections: 1. It is not from the section of all nerves that the decided effects on nutrition and secretion of which we speak result. The sensitive parts of the 5th and of the 8th are those from the section of which these effects (viz. on the eye, the lungs, and the stomach,) have been chiefly observed; and, even in the case of these nerves, it is only certain of the textures supplied by them which suffer from their section; chiefly the mucous membranes, hardly at all the serous membranes, or the skin or the muscular texture, which have their nerves from the same source. 2. Even in the case, of which much has been said, of the section of the par vagum suspending the functions of the stomach, the result of the experiment is not uniform; for, in the experiments of Leuret and Lassaigne on the horse, although portions of the par vagum on each side of the neck were cut out of the body, such a secretion of gastric juice nevertheless took place as was found to be effectual for the formation of chyme and of chyle from food taken after the section; and a positive result in such a case is, of course, of more weight than many negative results. And further, as in Dr. Wilson Philip's well-known experiments, the secretion of the stomach, when suspended by cutting the 8th pair of nerves, was restored by galvanism, unless we are prepared to maintain that the nervous influence essential to secretion is identical with galvanism, (a supposition which involves many difficulties, if not actual absurdities,) we must admit that no nervous influence transmitted along the nerves can be essentially concerned even in this secretion. 3. The effect produced on the nutrition and secretion, even of the parts most easily affected in this way, by section of their nerves, is not simple suppression, probably, in any case. In the case of the mucous membrane of the bronchi and lungs, it is always an *increase* of the quantity, attended no doubt with a change in the quality, of the secretion; and after the section both of the 5th and 8th nerves, as also in other cases where sentient nerves have been palsied, the ultimate result is not simple death of the part, but *inflammation*, the formation of various inflammatory products; and, if death ensues, it is death preceded and caused, in the usual way, by inflammatory action.* Such a *series of changes*, therefore, ensues in these secreting parts from the section of their nerves, as was observed by Bichat in experiments on the nerves of the testis, the only secreting organ, as he thought, which could be completely isolated from the larger masses of the nervous system by cutting its nerves. After the section of these nerves, he states that he could not observe that the secretion of semen went on; but the reason of its not going on he thought equally conclusive as if it had, against the dependence of secretion on nervous influence, for the testicle inflamed and *suppurated*—i. e. a *morbid secretion* could be established and maintained, notwithstanding that this supposed influence was removed; and, if so, the formation of a new product out of the blood, which is the essence of nutrition and secretion, must be within the ordinary operations of nature, independently of such influence.†

Again, the case repeatedly observed of the fœtus coming to the full size, with the usual variety of textures in its composition, without either brain or spinal cord, has always appeared to us a natural experiment, conclusive as to the principle that nutrition and secretion cannot be dependent on an influence generated in these organs, and transmitted along the nerves. It is said, indeed, by Mr. Travers and others, that in this case the ganglia, likewise supposed to be sources of nervous influence, exist in the body: but, without adverting to the cases on record, where these would seem to have been carefully looked for, and not detected,‡ we request attention to this fact. If the ganglia are the source of the nervous influence by which nutrition is effected, and of the effects produced on glands and on mucous membranes by section of nerves, are owing to the interruption of that nervous influence, then, if the ganglia and ganglionic nerves are left untouched, no such effects should result from injury of the brain or spinal cord. But it is well known that disorganization of a very small portion

* This is not duly considered by Dr. Marshall Hall, who says that when the 5th nerve within the cranium is compressed or destroyed, "the eye ceases to be nourished, and becomes destroyed." (*Lect. &c.*, p. 31.)

† See Anat. Gen., t iv. p. 604.

‡ See Elber De Acephalis, pp. 31, 35, 45.

of the spinal cord, palsying the lower limbs, has often led to alteration of the brain, and to inflammation and sloughing of the mucous membrane of the bladder, notwithstanding that all the ganglia and ganglionic nerves were untouched.* This we take to be clear proof that secretion and nutrition (just as was stated in regard to involuntary motion,) may be grievously injured by injury of parts of the nervous system, which are nevertheless unconcerned in the ordinary exercise of those functions.

On these grounds, we maintain with confidence that the inference attempted to be drawn from the effects of injury of *nerves*, as to the dependence of nutrition and secretion on an influence transmitted through them, is equally unsupported as that which was attempted to be drawn by Le Gallois from injury of the *spinal cord*, as to the dependence of the heart's action on an influence derived from thence.

The notion of a vivifying "nervous influence," like the ideal theory of metaphysicians, has become so interwoven with the speculations of some physiologists in this country, that we can hardly expect to see it confined within its proper limits in the present generation. But whenever this phrase is employed in reference to acts of organic life, its vagueness should be borne in mind; when it is stated, for example, by Mr. Mayo, that "parts *from which the nervous influence is withheld* are more disposed to inflame, and suppurate, and slough, than unhealthy parts;" we think it obvious, either that an "unphilosophical hypothesis" is proposed, or else that a very objectionable because theoretical expression is employed to denote what is simply and precisely expressed by saying that parts, the nerves of which are injured, and which are thereby rendered insensible, are so disposed to inflammation.

That the doctrine of a nervous influence essentially concerned in the organic life of animals, is *truly hypothetical*, and not adequately supported by observations confined to the effect of injuries of the nervous system, will perhaps appear more clearly, if we contrast it with another doctrine, allowed to be partly hypothetical, but proposed with some confidence as at least equally consistent with the facts, and much simpler and more satisfactory to the mind that contemplates it, viz. that the whole organic life of animals—i. e. every thing which goes on in them without the intervention of any sensation or other mental act,—may go on without the intervention of the nervous system, and stands in no relation of *dependence* to any changes in nervous matter; just as the corresponding functions of circulation, nutrition, secretion, absorption, go on in equal perfection in the lowest class of animals, where no nerves are detected, and in the whole vegetable kingdom, where there is no plausible reason for supposing that nerves exist; that the nervous system lives and grows within an animal, as a parasitic plant does in a

* See T. Stanley, in Med.-Chir. Transactions, vol. xviii.

vegetable; that with its life and growth certain sensations and mental acts, varying in the different classes of animals, are connected by nature in a manner altogether inscrutable to man; that the objects of the existence of animals require that *these mental acts should exert a powerful controlling influence* over all the textures and organs composing an animal; and that this influence is exerted by means of certain vital properties, with which the different parts of the nervous system are endowed, and that the effect of physical injury or disease, in many cases, is, *imitating the effect of the sensations and other mental acts,* to excite these endowments of the nervous system into action, and thereby to produce changes more or less exactly similar to those which, in the perfectly natural state, those sensations, voluntary efforts, or other mental acts are capable of producing.

That this statement of the office of the nervous system in the animal economy is the truth, we may assert without the slightest hypothetical assumption. That it is the whole truth, we admit to be still doubtful; but that anything more than this has ever been proved to be the truth, we think ourselves entitled to deny.

If our readers are as strongly impressed as we are with the importance of a right understanding of this preliminary question to the future progress of physiology, they will not think the time misspent which is directed to the attempt to adjust it. But we must now admit, that the judgment we form upon it is of less *pathological* importance than might at first be supposed; for all physiologists, even those who most strenuously oppose the doctrine of a *vivifying influence* necessary to the performance of the organic functions of the body, flowing from the nervous system,—admit that a controlling power over all these functions is possessed by some portion or other of nervous matter; i. e. that certain changes which take place in that part of the living body affect, in various ways, all these functions, and therefore that all external agents which are found to influence the organic functions *may* produce their effects through the intervention of the nervous system.

The reality of this *controlling influence* of changes in the nervous system is most clearly indicated, and the mode of its operation to a certain degree illustrated, by the effects of violent injuries of different portions of the nervous matter. The effects of such injuries on the actions of the heart, and on the circulation through the larger vessels, is best illustrated by the experiments of Le Gallois and of Dr. Wilson Philip taken together; and the most important general result of them is, the inefficacy, on the larger organs of the circulation, of any injuries inflicted on *small* portions of the nervous matter, in any part of the system; and, again, the powerful exciting or depressing influence exerted on the actions of the heart by injuries which extend at once to *any considerable portion* of the brain or spinal cord; those which primarily affect the cervical portion of the cord appearing to be, *cæteris paribus,* the most powerful, but by no means the sole causes of this kind which produce this effect. The application of this fact in physiology to the case in pathology of concussion (of the spinal cord, as it ought to be termed, rather than of the brain,) as distinguished from compression of the brain, requires no illustration. Nor does it appear unreasonable to deduce from this leading fact two important inferences: 1. That a great part of the use of the very complex system of the ganglionic nerves supplying the heart is to combine and concentrate upon it the influence of causes which act over large portions, or throughout the whole extent, of the cerebro-spinal axis; and, 2. That all causes which, although apparently applied to or originating in other parts of the system, suddenly and powerfully excite or depress the heart's action, if not carried to it by the blood, must in all probability affect it only by first affecting a great part or the whole length of the cerebro-spinal axis,—i. e. the origins of the sympathetic nerve. Thus we acquire the knowledge that all the exciting causes of syncope, all intense sensations and overpowering mental emotions,—all such injuries or rapid changes as, although apparently confined to individual and extreme parts of the body, affect the system in the way of a concussion or shock,—are naturally attended by changes in the nervous system of this extensive character, and affect the circulation of the blood by reason of this peculiarity. Many of the causes of sudden or violent death illustrate this point; and it is perhaps still better illustrated by a class of cases of apoplexy, first distinguished, we believe, by Dr. Abercrombie, in which there is sudden sinking of the circulation, from which the patient rallies, followed by uniformly fatal coma, and dependent on equally uniform extensive extravasation of blood. (See *Path. Researches,* &c. Part II. sect. ii.)

But an influence, equally resulting from changes in the nervous system, and probably of greater pathological importance, is illustrated by some of the experiments on injuries of the brain and spinal cord, particularly of Le Gallois, Flourens, and Chossat, in which it clearly appeared that, even when injuries of these larger masses of the nervous system failed to affect the heart's action, they always took effect on the flow of blood in the *capillary vessels,* and might often be so managed as to influence especially or wholly suspend the circulation, and all the subordinate vital actions, in those organs only which had their nerves from the injured portions of the cerebro-spinal axis.*

"Dans tous les cas," says Flourens, "la destruction complète du systême nerveux affaiblit tellement l'ensemble de la circulation, que quelque tems que la circulation vasculaire survive encore, la

* See Le Gallois' Experiments, &c., p. 120, 140; and Flourens' Recherches Exp., &c., p. 196.

circulation capillaire souscutanée est toujours presque instantanement éteinte." Again: "Lorsqu'une region determinée du systême nerveux est seule detruite, c'est toujours dans les seules parties correspondantes à cette région que la circulation se montre surtout affaiblie." As it is generally allowed that most diseases originate in the capillary vessels, and only secondarily affect the heart, the influence which these facts illustrate is probably more nearly akin to that which the nervous system usually exercises in exciting diseases, than that which is observed from such injuries on the motions of the heart.

Although, therefore, we differ from Mr. Travers as to the necessary dependence of the organic life on the nervous system, and although we think he certainly goes too far when he says that "this system forms the portal, as well as herald, of *all* diseased action; and that no altered action, of whatever description, can be instituted but by its medium;"* yet we agree to his assertion, that "the *influence* of this system (i. e. the occasional exciting or depressing influence, not the constant vivifying influence,) is universal, both by solid and fluid, throughout the body; and think him justified in asserting, or rather in assenting to the assertion of many previous pathologists, that, when *local injury* or local disease produces constitutional irritation, or, more simply, *general effects* on the living body, the nervous system is most generally to be regarded as the medium through the intervention of which these effects are excited.

Nor do we think that Mr. Travers attributes too much importance to this principle in pathology, when he contrasts it, in the following sentences, with those simpler and more tangible, but less fundamental principles, which are capable of demonstration in the dead body.

"Out of the debris of the dead subject, however accurately inspected, examined, and arranged, to attempt a solution of the great problem of living actions, and to build on such a foundation an edifice of pathology capable of self-support, is as injurious a fallacy, and scarcely less arrogant or absurd, than that of the Cartesian philosophers, who undertook, out of the depths of their anatomical sagacity, to make a man." . . "A very doubtful service would be rendered to science, if the analysis and classification of the various changes met with in dead bodies were to be employed as instruments for the synthetical arrangement and elucidation of the vital phenomena; in fact, for the natural history of disease." . . . . "Until we know more of the springs and sources of action,—the mutual relation, especially, of the nervous principle and the blood,—the cause either of irritation or inflammation will not be determined by inspecting the state and action of the different orders of vessels in irritated and inflamed parts, or by classifying the various changes of structure after death; the former the symbols of the morbid action, the latter its effects. That these, nevertheless, should be the objects of our careful

* Further Inquiry, &c., p. 438.

study, no one will deny; but let them not be made of undue importance, lest they mislead us, and we commit the anachronism of a theory explaining the actions of disease, before we know those of health." (P. 212—16.)

But, while we fully acquiesce in the truth and importance of these observations, we must admit that it is very difficult to acquire precise and definite information, and very easy to deceive ourselves by plausible but vague and indefinite speculations as to the intimate nature of diseased actions, when considered independently of, or antecedently to, the formation of diseased structures; and particularly as to the mode and extent of influence of changes in the nervous system in exciting such actions: nor do we find, in Mr. Travers's work, any facts to give additional precision to our ideas on this subject. The experiments above mentioned, although they put beyond all doubt the special influences of certain changes in the brain and spinal cord on the circulation in the capillaries,—certainly the primary seat of inflammation, of fever, of much functional and all organic disease,—yet hardly carry us farther than the general principle which might be inferred from other facts, (and particularly from the influence of mental affections in altering the capillary circulation in health, and in exciting or modifying such diseases,) of the *possibility* of all such diseases, whether general or local, being excited through the intervention of the spinal cord and nerves.

In so far as Grief, Fear, Anxiety, or mental depression of any kind, acts as a predisponent cause,—and in so far as Cold acts as an exciting cause of any such disease,—it must be through the nerves of the parts where the diseased actions begin that these causes produce their effect; and that effect is, to a certain degree, illustrated by the effects, general or local, of sensations and emotions on the flow of blood in the capillaries, and on the other vital actions there, in health. In so far, again, as impressions on one part of the body are effectual in exciting diseased action either in the heart or in the minute capillaries of another part unconnected with the former; as the effect is of the kind called sympathetic, and appears to be dependent, like other sympathies, on sensation, an influence of the spinal cord and nerves is clearly denoted, and is evidently analogous to that which the physical injuries inflicted in the experiments now mentioned exert. These are the cases to which Mr. Travers gives the names of Direct and Reflected Irritation. It is unnecessary to say, that this description applies to the usual mode of excitement of many inflammations, and of some other diseases, both acute and chronic; but it must be admitted that the kind of explanation thus obtained is very deficient in precision.

More specific information may be thought to be given by the result of experiments on individual nerves, particularly the 5th and 8th, of which we have already spoken. The alterations of secretion and nutrition, and the inflammations in the eye, the lungs, and the stomach, produced by section of these nerves, have been the subject of numberless experiments and of keen disputes, as is well known to all who are familiar with the writings of Wilson Philip, of Broughton, of Swan, and of Mayo, in this country, and of Magendie, Breschet, Milne Edwards, Brachet, &c. in France. But the question is still undecided in what manner the section of these nerves leads to the inflammation and disorganization of the textures; whether it acts directly as the exciting cause of these changes, or indirectly by arresting vital actions, which are naturally consequent on sensations, and by disposing the parts of which the nerves are cut to take on inflammation from other causes, which would otherwise be innocuous. Without pronouncing decidedly on this question, we may observe on the latter point, that if the section of the sensitive nerves of a part were the direct cause of its inflammation, we should expect to see inflammation in all parts of which the sensitive nerves are cut; whereas, the phenomenon in question is seen only in a few parts; and in those parts it originates and is chiefly seated in a single texture, viz. the mucous membrane: that membrane is distinguished from others in the body by its power of bearing the contact of air, of foreign substances, and of excretions elaborated within the body, with impunity. This power seems obviously connected with its vital property of throwing out, when irritated, a mucous secretion which protects it equally, as the cuticle protects the true skin; and this adaptation of the quantity of protecting mucus to the irritation which may act on a mucous membrane, may be very naturally supposed to depend on its sensibility, and to cease when its sensitive nerves are divided, and allow the mucous membrane to inflame and slough equally as a serous membrane would do from the irritations which, in the natural state, excite only a healthy action upon it. On this supposition, the inflammations in question depend, not simply and directly on the division of nerves, but on the action of the air, the food, the bile, &c. on mucous membranes deprived of their sensibility, and thereby in a great measure of their protecting mucus; and bear an analogy to the inflammations of the same membranes which frequently take place from deficiency of the mucous secretion, in cases of death by starvation, and towards the close of lingering and exhausting diseases.

On this last supposition, the inflammations of the part in question, excited by the section of their nerves, are by no means analogous to those excited through the intervention of nerves, by cold or other causes, applied at a distance, in the entire state of the body; but, on the contrary, they demonstrate that the morbid process of inflammation may be excited, run its usual course, and produce its usual effects, in a part when we have no evidence that any vital actions, strictly peculiar to the nervous system, are going on.

Another well-known cause, in which inflammation of the part supplied by a nerve is, in part at least, the effect of loss of the nervous power, is the case of a paralytic limb inflaming from the application of a degree of heat which would not injure a sound and sensible limb. Cases of this kind are quoted by Dr. Ley, in the work before us, (p. 357,) from the papers of Dr. Yelloly and Mr. Earle, in the Medico-Chirurgical Transactions. The only inference drawn by Dr. Ley from this fact is, that the nervous influence gives to the capillaries the power of resisting sudden alternations of temperature, which is lost when that influence is withdrawn; which, if it be meant for more than a mere expression of the fact, is both vague and theoretical. Probably the true explanation is merely this, that the limb being colder than a sound limb, has its temperature raised by the application of a given external heat through a greater number of degrees; and the effect of heat on vital action is in general proportioned, not to the severe temperature that may be applied, but to the degree of change of temperature which is undergone, as is seen, *inter alia*, in the inflammation of frostbite succeeding the restoration of the natural temperature to a limb which has been cooled much below it. If this be so, this effect of injury, or loss of function in a nerve, is likewise obviously *indirect.*

Dr. Ley has stated (p. 274 and 302,) we think quite correctly, that the coldness generally observed in a paralytic limb, and the corresponding symptom of increased heat in a limb, of which the sensitive nerves are much excited, as in neuralgia, ought not to be regarded as proofs that animal heat is evolved from the blood by the action of the nerves. He argues that, although the circulation continues in a paralytic limb, the nutrition and secretion are usually much diminished; and therefore that those changes in the arterial blood which, on chemical principles, are to be regarded as the cause of the evolution of heat from it, are defective. He does not seem to be aware that an elaborate investigation, by Dr. Chossat, into the effects of injuries of the nervous system on animal heat, gave this result, in perfect accordance with this view of the subject, that all those injuries, and only those injuries, which materially influence secretion and nutrition, influence also the evolution of animal heat.

The whole effect of increased excitement of the functions certainly belonging to the nerves, particularly to the sensitive nerves, in any part of the body, (as in cases of neuralgia,) in exciting the flow of blood to the part, raising its temperature, and often increasing its secretions and ultimately its nutrition, are well illustrated by the recital of various cases in Dr. Ley's work, (p. 301 et seq.) In particular, he gives four cases which show, what might at first be thought quite inconsistent with the facts above stated, that neuralgic pains and increased sensibility in a part, keeping up a constant congestion of blood in it, sometimes occasion it to inflame from slight causes, such as leechbites, and frustrate the cure of such inflammation.

These facts are important to our present purpose, because they show how much the actual circulation may be influenced by changes in the sentient extremities of the nerves, the effect of which is "retrograde along the branches of the arteries;" and this is obviously one of the chief causes of the want of harmony, so frequently observed, and so important to be understood, both in the healthy and diseased state, between the actions of the heart and the flow of blood in the capillaries of any one part of the system. But we may observe, although incidentally to our present subject, that Dr. Ley, as well as most other authors, use language which we hold to be demonstrably incorrect, when they say that the consequence of increased excitement of nerves is "a local *increased action of blood-vessels*," or "increased activity of the minute vessels of supply." The change on the vital condition of the blood-vessels, in such cases of locally increased determination of blood, is *diminution*, not increase, of their tonic constriction, the only kind of vital action of which they appear to be susceptible; and the only question is, whether this diminution, giving increased effect to the impulse from the heart, is adequate to explain the phenomena and varied effects of local determinations of blood, or whether such determinations are in part to be attributed to causes affecting the motion and distribution of the blood, but independent of any contractions of moving solids.

It must be admitted, however, that these examples either of injury or disease of nerves affecting the capillary circulation bear only a remote analogy to the more usual modes in which the diseased actions, primarily affecting that part of the circulation, seem to be excited: and we think it ought also to be stated that there is still much ambiguity as to the mode of operation of a very important set of morbific causes, viz. the Poisons, of which Mr. Travers (much too confidently, as we apprehend,) asserts, "that there is but one principle on which they operate, viz. the nervous system."

It is, indeed, we believe, generally admitted, when a poison acts with so much rapidity as the strong hydrocyanic acid, or when a mineral acid is taken in such a concentrated form as to disorganize the stomach, and kills more rapidly, without making its way into the circulation, than the same acid taken in a diluted form, and manifestly absorbed into the blood,—that the poisonous effect depends simply on an impression made on the sentient extremities of the nerves, and immediately transmitted to the larger masses of the nervous system, and thence to the circulating system, and which may be held to be analogous to a concussion. But, in the more common case, where the action of a poison applied to the surface of the body is slower, and where the experiments of Fontana, Brodie, Barry, and many others, show that its absorption into, and transport by the blood, is an essential preliminary to that action, what evidence have we that it is through the nervous system that it produces its fatal effects? Mr. Travers seems to think that, even in this case, the circulation of blood at the part is necessary, not as a means of transporting the poison into the system, but only as a necessary condition to the excitability of the nerves of the part, (see p. 396;) apparently not being aware of the experiments of Segalas on the effect of poisons inclosed in isolated portions of intestine, in which it appeared that, although the circulation at the part was left free, if the vein leading from it was punctured, so as to discharge its blood, (in which case it seems obvious that the nerves of the part must have remained excitable, although the blood passing through the part did not return to the heart,) the poison was equally ineffectual as when the vein was tied.* This experiment, we think, compels those who believe that the only action of poisons is through nerves, to resort to the supposition of Dr. Addison and Mr. Morgan, that the sentient extremities of the nerves *of the veins* are the part of the nervous system on which that effect is specially produced.

Although this last opinion is not quite in accordance with that which Mr. Travers maintains in the passage to which reference has been made, yet he holds this opinion also, and refers to the well-known experiments of Dr. Addison and Mr. Morgan in support of it, but especially to that one (an *experimentum crucis* in his opinion, but which he has not quite accurately described at p. 390,) in which a poison is included in a tube between two ligatures on a vein; the ligature *farthest* from the heart being removed, the poison, previously inert, *immediately* becomes active. But, although this result is certainly different from what might have been anticipated on the supposition of the circulation of the poison in the blood being essential to its action, yet we cannot regard it as a conclusion against that supposition, unless it were shown that the poison, when the ligature above it is removed, and when it mingles itself with the stream of blood in the vein, does not taint this blood as far back as the next anastomosing branches, and so make its way forwards to the heart. That this is not the effect of removing the farther ligature is not shown by these authors; and their other experiments in favour of their peculiar doctrine of the mode of action of poisons, we have no difficulty in pronouncing to be inconclusive.

When, on the other hand, we consider that most poisons manifestly affect the vital constitution of the blood; that they have appeared in experiments on the heart, and on the stomach and bowels, when still acting after their removal from the body, to affect them nearly in the same manner as when their connexion with the nervous centres was entire; and, still more, that they *act powerfully on vegetables;* we confess that we are by no means

* See Journal de Physiologie. 1822.

satisfied with the evidence of the theory which would resolve the whole *modus operandi* of poisons into an impression on the nervous system; and considers it much more probable that, when they circulate in the blood, they directly affect *all* the living textures and all their vital endowments,—not the nervous system only, but the muscles, the glands, the organs of assimilation and nutrition, and the blood itself; some of them, however, exerting a special influence on one of these parts, and others on another.

We think it the more important to state these considerations as to the action of poisons, as that action is clearly more analogous than anything else in relation to the mode of excitement of some of the most formidable diseases, on which Mr. Travers pronounces an equally decided opinion. He applies the term *constitutional* to erysipelatous inflammation, to gangrenous inflammation, and in general to those inflammations which show the "disposition to appear independent of injury, to relapse or reappear in the same organ, to spread by continuity, to appear in various parts or organs at the same time, or consecutively in different textures, premising the same characters as tubercular deposit, or suppurative inflammation, or erysipelas; to pass rapidly from the state of phlegmonous inflammation into erysipelas or gangrenous inflammation." And, in regard to "secondary inflammations, i. e. inflammations partaking of the same character in distinct parts of the body," he says that they "show more than any other the influence of the constitution on the local action;" and that he "cannot attribute any of these to absorption or the introduction of pus into the blood, but to the principle of morbid irritation, extending to the system at large, which was at first confined to the part." (P. 223.) These expressions are not so definite and precise as could have been wished, but they seem plainly to indicate an opinion that several of the varieties of inflammation which are now usually called *specific*, and particularly erysipelas, differ from the more ordinary form of inflammation, not in their external cause, but merely by reason of peculiarities of constitution of the person affected; and that, when the singular phenomenon is observed of purulent deposits, preceded by slight and brief inflammatory symptoms only in the part where they take place, but preceded by the gradual formation of large quantities of pus in distant veins, or in other parts of the body, this is by no means to be ascribed to any absorption and circulation of purulent matter in the blood, but only to a "principle of morbid irritation," acting (we presume he would add,) in that *primum mobile*, according to him, of all vital action, healthy and morbid, the Nervous System.

Now, in opposition to this, we think it may be maintained, almost with certainty, that the epidemic extension of cases of erysipelatous and gangrenous inflammation, particularly in hospitals, and their occurring in persons of all possible varieties of previous health and constitution, are sufficient to show that their peculiarities often depend, not on the constitution in which they appear, but simply on the cause from which they arise; a cause often obscure, but sometimes as distinctly conveyed from one individual to another as smallpox or measles is. And, in regard to the theory of the secondary purulent deposits, when we observe the extraordinary rapidity with which they form, (e. g. within the cavities of joints,) and recollect that the purulent matter has been often detected in large quantities in the veins leading from suppurating surfaces, we cannot hesitate to express our belief that it is the presence of this morbid matter in the circulating blood which determines, in such cases, this peculiar effect of the secondary inflammation, wherever or however it may be excited.

It is very possible that great part of the symptoms which distinguish a specific (e. g. erysipelatous) inflammation, may be really owing to changes in the condition of the nervous system; and we think Mr. Travers established in his former work, more distinctly than any previous author had done, the important principle that certain injuries, primarily affecting the nervous system, are naturally followed by a form of fever which is typhoid rather than inflammatory, and very analogous to that which so frequently attends the specific inflammations now in question. But we adhere to the belief that the most essential and important parts of the pathology of many such cases of specific inflammation are, the peculiarity of their external cause, and the change effected by that cause on the constitution of the blood; and that the affection of the nervous system is only a part of the effects which that external cause, introduced into the circulating system and tainting the blood, produces on different textures of the body.

We think it evident, from what has now been stated, that the share which the nervous system has in producing those diseased actions which show themselves most distinctly in the Organic Life,—in the function of circulation, and in the vital actions of which the capillary vessels are the seat,—still requires much careful and discriminating investigation, and is by no means satisfactorily expounded by the sweeping assertion of Mr. Travers, that the "nervous system forms the portal as well as the herald of all diseased action."

II. In proceeding to enquire into the exact state of our knowledge as to the pathology of the strictly Animal Functions,—and first as to those modifications of the animal functions which depend on changes confined to the *nerves*,—we enter on ground of more limited extent and less general importance, but fortunately admitting, in the present state of physiology, of the establishment of more definite and satisfactory principles, and indeed of a precision of knowledge which was lately thought unattainable; and we avail ourselves, with much satisfaction, of the work of Dr. Ley as a guide; because, although many facts on this subject are contained in the writings of Sir C. Bell, Dr. Abercrombie, Mr. Swan, Mr. Mayo, and others, the general principles which these facts may be thought to establish are more distinctly laid down in his work than in any of the others.

The following are the propositions relative to injury and disease of nerves which Dr. Ley regards as established:

"1. That, if injury or disease affect the trunk of a given nerve, the principal effect will be observed at *the remote extremity* of its filaments. 2. That *all* the branches proceeding from such common trunk will have a *similar disturbance* of function, from the same morbid impression. 3. That the morbid affections of nerves resolve themselves into those of *excitement* and those of *defective energy*, each promoting peculiar and definite symptoms. 4. That excitement may be the result of *mechanical impulse*, of *vascular congestion* and irritation, of *inflammation*, of *structural disease*, and sometimes perhaps of *simple functional disorder:* and 5. That diminished energy is generally the consequence of some *extraneous pressure* on a healthy nerve, or of *atrophy*, either primitive or secondary, of the nerve itself." (P. 286.)

On most of these points, however, some limitation of the statements is necessary, and some difference of opinion may exist.

1. Of the first statement there is this important limitation, that it is only in the *healthy*, not in the diseased state of a nerve, that the effects of injury, or of fresh disease, are confined to its extremities. Dr. Ley's own work, as well as the others before us, contains many cases in which *extensive* pains or *general* spasms resulted from *local* disease of individual nerves of some standing; and Mr. Mayo has stated, in connexion with this, and illustrated by cases, the important practical principle, that when neuralgic pains *immediately* succeed an injury or wound,—such as that from bloodletting,—they may be arrested by dividing the nerve which has been wounded, above the part injured; but that, if the pains commence *after the cicatrization of the wound*, the chance is that the disease excited in the nerve has extended too far to be relieved by that operation. (*Outlines*, p. 134 et seq.) Dr. Ley states, however, that, "in the natural and healthful state, nerves are probably media of transmission only,—*les agens de la transmission des impressions;*" and this appears to be so much the case, that it is well known that such compression of a nerve as excites no pain may excite the sensibility of all its parts below the point pressed. Still the fact is not correctly expressed by saying that sensitive nerves, in the healthy state, "are in themselves little sensible:" the proper expression seems to be, that they are composed of filaments, the sensibility of which is strongest, and for useful purposes is almost exclusively excited, at their extremities.

It is unnecessary to quote illustrations of the well-known fact that the pain resulting from irritation of a sensitive nerve is referred to its extremities, and that the spasms excited in muscular parts by irritation of a nerve are likewise at the extremities of that nerve; nor do we think that there is any such difficulty as Dr. Ley states in reconciling this fact to Sir Charles Bell's principle of a nervous circle, connecting the voluntary muscles with the brain. Every nervous filament, according to that principle, is capable of transmitting impressions in one direction only, and has no power to "act both to and from the sensorium." Dr. Ley thinks that this rule is transgressed when "an impression is made upon a nerve, and sensation is experienced at a point more distant from the brain than the seat of the impression;" and proposes different modes of reconciling the fact to that principle. But it appears to us that the fact is quite in accordance with the principle. The impression is made on a nerve somewhere in its course; but it is made on the same nervous filaments which terminate at the sentient extremities of the nerve, and which are used in the natural state only to transmit impressions up to the medulla oblongata from these sentient extremities. When an impression is made at an unusual spot in the course of the nerve, it is still transmitted *upwards,* and, coming through the same filaments, it is *referred* by the mind to the same parts as that which was wont to be transmitted from a greater distance; but there is no transmission *downwards* by that nerve.

When an impression is made on a sensitive nerve consisting (as is now ascertained to be common,) of different filaments with different endowments, it does not always affect all those filaments, and therefore does not always excite the sensations which are usually referred to that nerve. But, if a nerve be forcibly pinched or compressed in any part of its course, it may be presumed that every filament which it contains must be injured. This is probably the explanation of the curious fact observed by Dr. Marshall Hall and Mr. Broughton, in experiments on the ass, that irritation of the par vagum in the neck with a needle excited no such instinctive effort as to indicate any peculiar sensation; but compression of that nerve with a pair of forceps was followed by a cough and effort of deglutition, implying apparently the production of sensations which, in the natural state, result only from impressions on the mucous membrane of the air-passages and pharynx.

2. In illustrating the proposition that "all the branches proceeding from a common trunk will have a similar disturbance of their function from the same injurious impression," Dr. Ley has, we think, omitted a consideration of some importance. He says that the apparent exceptions to this rule are to be explained only by filaments of sensation and of muscular movement being bound in the same sheath, and nevertheless capable of being differently or even oppositely affected; and it is in this way only that he can explain the occurrence of "long-continued, periodical, agonizing pain, while the muscles supplied from the same source were become paralytic and shrunk." In this case, indeed, we may suppose that the motory filaments of a set of compound nerves have lost their power, while the sensitive have been excited to increased action. But this explanation does not apply to the case which we have repeatedly seen, and which he has himself elsewhere described, of severe pain distinctly referred to a part of which the *sensitive* nerve (e.g. the 5th nerve, as supplying the face,) is palsied. Here the explanation obviously is, that the same injury or disease of one *portion* of a nerve which transmits fallacious impressions upwards, and *excites the action* of the portion of nerve above it, prevents the transmission of natural impressions from below, and so *suspends the function* of the portion of nerve below it.

3. On the division of the morbid affections of nerves into those of "excitement and of defective *energy*," we would only remark, that the last word is objectionable, because in the minds of many it implies a theory, and that we would greatly prefer the terms excessive or deficient *action*. Dr. Ley accurately describes the peculiarities of those pains which proceed from disease of sensitive nerves; their being "sometimes periodical, much more frequently paroxysmal; their occasional concurrence with convulsive starting of muscles;" their returning at irregular periods, and from very slight or unobserved causes, and their "fearful severity." He is right, we think, in excluding from this general description the characters—often given, but, except in the case of such pains in the extremities, not so often distinctly recognized,—of neuralgic pains *following the course* of nerves. But he illustrates at some length, as has been already noticed, the local determination of blood to the part affected,—the sense of heat,—the increased sensation in some cases, increased nutrition in a few, and proneness to inflame from slight causes. The greatest deficiency that we observe in this part of his work is, that he does not enter into any details as to the parts of the body in which these pains, dependent on diseases confined to individual nerves, are most generally observed. An accurate analysis and abridged description of cases of severe suffering, ascertained to be dependent on this cause, in different parts of the body,—in the face and head, in the neck, the sides of the trunk, within the thorax, the abdomen, and pelvis, as well as in the extremities,—would be of very considerable value, particularly to young practitioners, who are apt to confound such cases either with inflammation of other textures, on the one hand, or with more dangerous organic diseases on the other.

The intermitting or remitting character of diseases strictly seated in the nerves is justly stated as an important characteristic, referrible only to that general law of nervous action, both healthy and diseased, (evidently one of the points of analogy subsisting between it and muscular contraction,) that all such action naturally alternates with periods of inactivity, and all unusual increase of it with intervals of diminished power.

4. It will be allowed on all hands that "mechanical impulse, or other local stimuli, vascular congestion, inflammation, structural disease, and functional disorder," are all causes from which excessive excitement of nerves and painful or spasmodic diseases may originate; but the discrimination of these causes in individual cases is often difficult, and the real cause irritating a nerve, where it can be ascertained at all, is often ascertained only after death.

Among mechanical causes of excitement of nerves Dr. Ley enumerates, as has been common, "forcible distention," but he judiciously observes, and supports his opinion by reference to experiments of Swan, Bichat, and Bourdon, that paralysis is infinitely more common as a consequence of *mere stretching* of a nerve than excitement, and that the pain often observed from extension of nerves is probably chiefly owing to the inflammation and ulceration which result.

We are accustomed to speak so vaguely and confidently of "vascular congestion and irritation," as a cause of increased action of any part of the nervous system, that our readers may be surprised to find only a single *unequivocal* case recorded by Dr. Ley, from Bichat, of severe pain of a nerve (the sciatic) proceeding from this cause; the nerve being encompassed and penetrated at its upper part by a little plexus of varicose veins.

Dr. Ley has taken much pains to illustrate the subject of inflammation of nerves, or their neurilema, in its first stage, as a cause of morbid excitement of their functions; although, in the advanced stage of inflammation, when the nervous matter is either softened and disorganized or compressed by effused lymph, it is naturally to be expected that its functions will be suspended. But, among his statements on this subject, we meet with several, of the accuracy of which we are by no means satisfied.

He points out no means of judging whether increased excitement of a nerve results from inflammation, excepting only the pain and tenderness in the course of the nerve affected, which of course must frequently be imperceptible, from its internal situation; but he enumerates distinctly the different structural changes consequent on and indicating inflammation, which may be ascertained on dissection. Among these he specifies particularly "*permanent redness*, observable soon after death." Now, we must be permitted to deny that any such alteration of colour, and of the quantity of blood in the minute vessels of a portion of nerve, or even ecchymoses in its substance, *without any appearance of inflammatory exudation*, is a true indication of inflammation. At the time of the minute investigation of the morbid appearances in cholera, we saw so many examples of these appearances in various nerves, in persons dead both of that and of other diseases, and whose symptoms were known, that we are confident that, if they indicate inflammation at all, it must be inflammation which neither produces excitement of any of the known functions of nerves, nor gives any other signs of its existence during life.

Of the exudation of lymph on the membranous coating of nerves, of the varieties of hardening and softening of the nervous substance itself, and of the suppurative inflammation and ulceration of nerves, he gives various examples. But we think he lays it down too broadly that the natural effect of the inflammation which effects all these changes is *excitement* of the functions of a nerve, and that it is only when there is such an effusion of lymph as to "*strangle* the nerve" that loss of power is to be expected. At least, in the case of nerves of motion (e. g. the portio dura,) we are confident, from the rapid occurrence of the palsy after inflammation (apparently rheumatic) has attacked the nerve, and from the nearly equal rapidity with which, in some cases, that palsy has abated; that the loss of power of the nerve, like the loss of power of muscular fibres affected with inflammation, has occurred in a very early stage, and certainly when the nerve could not have been "strangled" by effused lymph.

He is at pains to enumerate the *causes* from which inflammation of nerves or their coverings may result: he illustrates the efficacy of cold, of exposure to air, and quotes with approbation the opinion of Messrs. Langstaff and Swan, that the chronic inflammation and bulbous enlargement of the ends of the nerves of stumps, so often attended with frequent and acute paroxyms of pain, are frequently the effect of exposure to air, and may be prevented by drawing the nerves beyond the surface of the stump at the time of amputation, and cutting off such portions of them as may secure their receding beyond the reach of atmospheric influence. He then quotes several cases to show that where a nerve has been injured in any way, as by puncture or ligature, and afterwards becomes the seat of violent paroxysms of pain, these are generally to be ascribed, not to the original injury, but to a chronic inflammation of the nerve or its membrane; of which the injury was one cause, but not the sole cause. Most injured nerves, after venesection, for example, according to the statement of Mr. Abernethy, "are made troublesome by using the arm too soon, and bringing on inflammation; for I have never seen any bad consequences in those patients who have been unable to do anything."

So also, when unusually intense pain attends simple inflammation, ulceration, cancer, or the growth of a tumour, he considers the cause to be, not the mere stretching or compressing of the nerve, (of which he thinks the more probable effect would be diminution than excitement of nervous action,) but extension of the inflammation to the nervous matter itself, or its immediate covering; and he adduces several ingenious arguments to show that, in a case of Sir B. Brodie, the stretching of two branches of the lumbar nerves over two enlarged lymphatic glands, " like the strings passing over the bridge of a violin," was not an adequate cause of long-continued pain, and of "repeated attacks of intense suffering, attended with violent spasm of the muscles of the thigh;" but that these symptoms depended on the nerves partaking of the diseased state of these and other textures about the groin.

He applies the same principle to the case of aneurisms, and various other tumours growing in the immediate neighbourhood of nerves, and often causing the most intense suffering, sometimes even death itself, *per acerbissimos cruciatus*, by pressing or stretching, and, as he supposes, exciting inflammation, and ultimately ulceration, in these nerves. He allows, indeed, that tumours situated on nerves, (neuromata,) or the painful subcutaneous tubercles of Mr. Wood, which he supposes always to involve nervous fibrils, may cause intense pain by acting merely as mechanical irritants, like the needle or probe of the experimental physiologist. But he evidently inclines to the belief of chronic inflammation gradually excited in the nerve in every such case; and, although he admits that great excitement of the functions of a nerve, acute pain, or violent spasm, *may* result only from functional derangement, yet he seems much disposed to think that in all such cases some tangible disease would be discovered, if the nervous filaments were followed up through their bony canals, in a way in which M. Laennec has observed that "hardly any case has been examined."

Now, it is not a fair and complete statement of this question to say that the prevalent belief of neuralgic pains being in general essentially distinct from inflammatory pains, is grounded only on the negative fact, that no marks of inflammation have been found on dissection in cases where such pains have long existed. The belief rests, besides, on many positive facts in the history of such cases; on the suddenness of the attacks of the paroxysms of intense pain; on the completeness of the intermissions and maintenance of the general health during them; the long duration of the disease, the experienced frequent inefficacy of antiphlogistic means, and the occasionally signal efficacy of means (such as quinine in the hemicrania,) which have no powers over inflammation. Some of these considerations, indeed, further prove that, even when chronic alteration of structure in nerves or their neighbourhood is attended with neuralgic pains, these tangible and permanent diseases are not the *sole* causes of the symptoms, any more than a tumour in the brain can be the sole cause of fits of epilepsy; but must be combined with some more acute and more transient diseased action, to which, in fact, it may be regarded as standing in the relation of a great and permanent predisposing cause. Such diseased action frequently results in part from chronic alteration of structure, and is frequently also in part excited by inflammation, and admits of occasional and partial relief from the remedies of inflammation; but it may exist independently of either the one or the other: it is confined to the nervous matter, is known to us, like the healthy changes in that matter, only by its effects, and is therefore termed, with strict propriety, functional disorder of nerves.

5. After concluding the subject of morbid excitement of nerves, Dr. Ley goes on to treat of "defective nervous energy," or defective action of nerves; stating, first, the effects of their deficiency in the vital actions of a part,—not merely the diminution or loss of sensation or of voluntary power, or of both, but likewise the diminished nutrition, the diminished power of generating heat, and likewise (what we apprehend to be the result of the habitually low temperature thus produced,) the increased tendency to inflammation from heat; and, lastly, the "withering of the nerves themselves," more remarkably observed, we believe, in the case of the optic than of any other nerves, and affording another plausible ground for conjecture that the imperceptible actions which take place in nerves are in some degree similar to the perceptible actions of muscles.

He then proceeds to illustrate the principle, which he considers as an important step to more precise knowledge on this subject, that simple pressure on a sound nerve is always followed by more or less of *loss of the nervous power*, and that it is not by reason of any change in the degree of pressure, but only by reason of the inflammation, or other disease, affecting the nervous matter itself, which it may excite, or on which it may supervene, that this cause can ever produce *excitement* of any of the powers of nerves.

"Such contrary effects as pain and convulsive movement, on the one hand," says he, "and impaired sensibility, with or without defective muscular power, on the other, are in reality, as they might be expected to be, the result of causes differing rather in essence than in degree; and, while the former of these effects are the consequence of the various changes of excitement already considered, the latter are the result commonly of pressure." (P. 382.)

Indeed, atrophy of the nerve, "primitive or secondary," is the only other cause of diminished nervous energy which he admits.

But we must be allowed to doubt whether the change in the condition of the matter of a nerve on which the loss of its power depends, is always to be distinguished from that on which its excitement depends in this simple manner. We have already stated that there are examples of inflammation, at least of motor nerves, in which they are completely palsied; while there is neither evidence nor probability of their being compressed or "strangled" by effused lymph. On the other hand, what is the "mechanical impulse," in the experiments of physiologists, and in injuries and sometimes in diseases of the human body, already stated as a cause of excitement of nervous action, but pressure? What is the compression of the par vagum, in the experiments of Marshall Hall and Broughton, by which the efforts of inspiration and of deglutition were excited, but pressure; and this on a sound nerve? It is plain, indeed, that a certain degree and a certain duration of pressure on a nerve destroy its powers, and that a more rapid or more frequently repeated pressure may excite its action only by causing it to inflame; and the distinction of these two cases is accurately stated by Dr. Ley: but, when he says that mechanical impulse on a sound nerve is a cause of excitement, and that pressure is a cause of palsy, we do not see that he differs materially from other pathologists whom he professes to refute, and who have taught that a certain degree or a certain duration of pressure excites a nerve, but that a greater degree or duration of pressure, acting on an uninflamed nerve, palsies it.

We think it best not only to admit, but expressly to avow, that the condition really essential either to excitement or to loss of power in a nerve is unknown to us, perhaps inscrutable by us, simply on account of the very minute structure of the nervous matter; and then to state the different external causes or antecedents by which each of these morbid states has been observed to be produced, without dogmatically asserting that either of those conditions is an uniform and necessary consequence of any one of those antecedents.

Dr. Ley further illustrates the pathology of nerves by an elaborate discussion of the "functions and attributes, in their natural and diseased conditions," of the most singular and complex in its endowments of all the nerves, viz. the Par Vagum; beginning this part of his work by a distinct statement of the distribution of the different branches of this nerve, and of the functions of these branches, so far as they are known. Of the physiological view of the larger branches he gives, we believe, a very accurate account; but we must protest against the vague and unsatisfactory statement, at p. 413, of the use of the smaller "filaments of communication" between this and other nerves of the neck. The connexion of these filaments, he says, "with the lingual nerve is to associate the respiratory organs with speech and deglutition; with the cardiac plexus, to associate them with the heart's action; with the facial, for the purpose of connecting them with the muscles of expression; with the three upper cervical nerves, that the respiratory function may be partially under the influence of volition; and with the sympathetic, that secretion may be insured:" every word of which statement is hypothetical, and, as we firmly believe, erroneous; the greater part of it proceeding on the fundamental assumption that sympathetic actions may be explained by nervous connexions, the fallacy of which was clearly pointed out, "sixty years since," by Whytt, Monro, and Haller.

It is clearly proved, however, by experiments on animals, especially those of Cruveilhier, that a convulsive cough may originate from irritation of the par vagum, the irritation apparently *imitating in the sensation it excites, and therefore in the effect it produces, those natural irritants of the air-passages* by which cough is excited; and Dr. Ley quotes various cases of injury, or at least probable injury of this nerve, in surgical operations, or in diseases where this symptom has been urgent and unexplained by any morbid changes in the air-passages or lungs. We have ourselves known examples, not only of cough, but of paroxysms of dyspnœa, connected with inflammatory effusion, either at the origin of the par vagum or at the passage of those nerves out of the skull which are ascribed to this cause; and we agree with Dr. Ley in thinking that, "in nervous sensitive patients, and especially in girls about the period of puberty, violent, deep, sonorous, paroxysmal cough, aggravated rather than improved by depletion, and cured by chalybeates," must in general be owing to simple functional disorder of the par vagum: and in so far as asthma and hooping-cough are truly spasmodic diseases, they are obviously dependent on spasms of parts which are moved by the motor branches of these nerves, and are therefore dependent either on functional disorder or on a certain degree of structural change in them. He quotes cases also in which severe dyspeptic symptoms probably resulted from disease affecting the lower branches of the par vagum; and we have ourselves seen one remarkable case, in which a slight morbid deposit, lying close on the œsophagus, just where it penetrates the diaphragm, had been connected with symptoms so closely resembling those of organic disease of the stomach as to have led different experienced practitioners to consider that disease unequivocally indicated. But Dr. Ley admits that "the symptoms which result from an excited state of those branches of the pneumo-gastric nerves which are distributed on the stomach cannot yet be stated with precision."

The effects which result from the division of the par vagum, and consequent loss of nervous action in its branches which supply the lungs and stomach, have been the subject of numberless experiments; and we have already adverted to the obscurity which still attaches itself to any explanation of these effects. On this subject we have only to remark here, that Dr. Ley very properly refers to the decisive experiments of Breschet and Edwards, and of Brachet, as proving that the peculiar sensations of the stomach and lungs are lost, (although, no doubt, other morbid and fallacious sensations are substituted,) and the peculiar motions of the œsophagus and stomach, and of the bronchi and back part of the trachea, suspended, in consequence of the section of these nerves; and we very much doubt the necessity of going farther than these acknowledged facts to explain the disturbance of the functions of the lungs and stomach which naturally results.

Of the effect of the loss of nervous action in the branches of the par vagum supplying the larynx,—i. e. the superior laryngeal nerves, by which the arytenoid cartilages are approximated, and the recurrent nerves, by which they are held asunder,—we are now very accurately informed, both by experiments and by pathological observations. The experiments of Le Gallois, of Magendie, and of

Bourdon, have distinctly shown that, where the recurrent nerves are cut, the glottis is very apt to be spasmodically closed, and the power of antagonizing the muscles which close it being lost, asphyxia very frequently results; that where the superior laryngeal nerves are cut, the muscular power of closing the rima glottidis, and probably the sensibility also just at that spot, are lost, and foreign substances are very apt to fall into the larynx, and excite, when there, vehement cough and suffocation; and that, when the par vagum is cut above the origins both of the superior laryngeal and recurrent, the danger is much less than when it is cut between the origins of these two, because, "both laryngeal nerves being deprived of energy, the chink remains immoveably open."

It is, as our readers are aware, precisely in conformity with these facts that Dr. Ley endeavours to prove that, in the "crowing disease" of children, and in the "roaring" or "cornage" of horses, the recurrent nerve on one or both sides is compressed, (generally by enlarged bronchial glands,) and has lost its power; the consequence of which is, that, whenever the superior laryngeal nerves act with energy, as in a fit of coughing, or in various other circumstances of excitement, the glottis is spasmodically narrowed for some time; whence arise "paroxysmal difficulty of breathing, with a loud sonorous inspiration, not observable in general when the body is at rest, but readily excited by any violent exertion." And, without presuming to deny that this kind of spasm at the glottis may take place while the recurrent nerves are healthy, we are satisfied, both from his statements and from some personal observation, particularly in cases of aneurism, that it is very frequently dependent on the loss of vital power from compression in these nerves. Nor do we think it at all unreasonable to conjecture, as Dr. Ley has done, that the peculiar sound of the hooping-cough may depend merely on a partial loss of power in the recurrent nerves, from the pressure of enlarged lymphatic glands, either within the thorax or in the neck.

III. The Pathology of the Spinal Cord, as illustrated in the different publications before us, presents no difficulty or obscurity, in so far as it is illustrated by what has been now stated in regard to the diseases of nerves. It is liable to alteration of its own substance from inflammation, terminating in softening, in decided suppuration, or in hardening, according to its character or degree; and in some cases it has been found in a state of complete atrophy, without obvious cause. It is likewise exceedingly liable to alteration or destruction of its vital powers by pressure; the prevention of which is obviously the main object of the essential peculiarity of the structure of all vertebrated animals, but which nevertheless frequently occurs from affusions of serum or of blood in the spinal canal, from inflammation, chronic thickening, or ossification, or the growth of tubercles, of hydatids, or fungoid or other morbid deposits on the membranes enveloping the cord, or from disease of the bones surrounding them. The nervous matter may be affected, in these cases, either by extraneous pressure or by extension of disease from the adjoining textures. From these causes we may expect, in conformity with what has been stated as to the diseases of nerves, to find the symptoms of excited, or of diminished or suspended nervous action in the parts supplied by the spinal nerves, variously blended in the progress of these diseased states of the cord; and these accordingly are found in the accurate epitome given by Dr. Abercrombie of the symptoms which may induce suspicion of incipient disease of the spinal cord.

"Weakness, numbness, or convulsive affections of any of the limbs; spasmodic starting of the limbs, occurring chiefly during the night; loss of the full power of the muscles, exemplified on attempting such motions as are required in running or leaping, numbness along the margin of the ribs, and a peculiar oppression and tightness across the region of the stomach; various affections of the breathing, difficulty in discharging the urine and fæces, or difficulty in retaining them." (*Abercrombie*, p. 381.)

He adds, that
"one symptom which should always be contemplated with much suspicion, is a feeling of pain and tightness, or constriction, along the margin of the ribs, as if a tight band were passed across the stomach, generally accompanied with a feeling of distention in the lower part of the abdomen, as if the bowels had in part lost the power of propelling their contents."

We have looked over his numerous cases illustrating the varied combinations and successions of these symptoms, in the view of ascertaining whether the principle which Dr. Ley attempts to establish in regard to nerves is applicable to the spinal cord,—i. e. that excitement of the nervous power depends on the progress of disease affecting the nervous matter itself, and that loss of power depends either on atrophy or extraneous pressure; but we do not find that any such distinction is uniformly observed. In cases where, from the appearances on dissection, we should say that the cord had been subjected to pressure only, and even to gradual pressure, as from effused serum, there appears to have been violent pain and convulsion, (as in Case 149, at p. 356, and the case from Morgagni, at p. 357-8, third edition;) and, on the other hand, in some of the cases where the substance of the cord had been either softened by suppuration or gradually hardened to the consistence of cartilage, there appears to have been no indications of excitement, and no symptoms but those of gradual loss of power. (See cases at p. 354 and 5, and at 365.)

Indeed, when we take a general view of those diseases of the nervous system in which its larger masses are primarily affected, but where the chief symptoms are in the voluntary muscles, it is impossible for a moment to suppose that a line of distinction between cases of excited and cases of diminished or suspended action can be drawn either from appearances on dissection or from a knowledge of the mode of operation of the causes of these diseases. When we know that the violent spasms of tetanus or of hydrophobia, or of certain cases of concussion, and that the total loss of voluntary power over most or all the muscles of the body, (as in Cases 144, 5, 6, 7, of Dr. Abercrombie's collection,) may take place without either perceptible lesion of the nervous structure or encroachment on the nervous substance in any part of the body, it is in vain to deny that the essential conditions of the most intense diseases of the nervous system are certainly unknown to us, and probably inscrutable by us; nor need there be any scruple about making this avowal, when we remember that the essential conditions of the healthy agency of nervous matter are equally unknown, that the closest microscopical inspection detects no difference whatever between a nerve which is exciting by an action in its interior, the most intense sensation, or the most violent muscular spasm, and one which is in a state of absolute inactivity. All that we can pretend to do, in laying down the pathology of the nervous system, is to describe and arrange the alterations of its functions which actually present themselves in practice; and then to state the causes, external or internal, which have most generally been observed to precede them, and the alterations of structure which have most generally been found to attend them. The diseased states, as well as the healthy actions of the nervous system, are known to us only by their causes and their effects, not in their own nature.

These observations apply to the diseases of the spinal cord only in common with all other parts of the nervous system, and to the spinal cord considered as the largest of the nerves, transmitting impressions upwards and downwards. The question, what diseased states of the animal functions originate or reside primarily in this portion of the nervous system, admits, no doubt, of a more definite answer than it has yet received. We have now good reason to believe that various diseases and symptoms of disease, which are in common language ascribed to the brain, really result from changes, whether functional or organic, confined to the spinal cord; or, in the language of Dr. Wilson Philip, are affections of the nervous, not of the sensorial functions. Thus, when concussion of the brain, or of the body at large, suddenly depresses the heart's action, enfeebles the pulse, and chills the surface, the experiments of physiologists leave little room for doubt that it is through the spinal cord rather than the brain that these effects are produced. So also, when the heart is suddenly weakened by loss of blood in the erect posture, the common expression, that the heart suffers from the sudden impression made on the *brain*, is probably incorrect; as the anatomical connexions of the heart are with the spinal cord, and particularly with its upper part, the medulla oblongata*

and cervical portion of the cord, not with the brain; so also we have reason to believe that it is by the impression made *there* that the effect is produced on the heart.

Dr. Marshall Hall has stated several facts which prove, more distinctly than any others which we know, the dependence of various spasmodic diseases on the spinal cord rather than the brain. This has often and naturally been supposed as to tetanus, where the excited action of the voluntary muscles is so great and general as to denote the affection of a central and common source of their vital power, and yet the functions proper to the brain are untouched; but the following fact is more distinctly in point than any reasoning.

"Tetanus may be produced at will in the frog or salamander, by applying strychnine to the skin. If the head be removed, the frame is still tetanic; if any portion of the spine, if even the tail of the salamander be separated, it exhibits all the phenomena of perfect tetanus. *These cease on destroying the caudal portion of the spinal marrow by means of a fine needle.*" (*Lectures on the Nervous System*, p. 42.)

That convulsion is strictly an effect of change in the spinal cord, and that any injury or disease of the brain produces convulsions only in as much as it affects the spinal cord in a peculiar way, may also be deduced as an inference from known facts, but is more directly established by the following observation:

"The usual mode of killing sheep is, first to divide the spinal marrow, and then to open the large vessels. At my request, not only the spinal marrow but the entire neck was divided, and the head separated from the body, with the exception of the skin; the blood-vessels were then divided, and I watched the effect of the loss of blood. After a certain hæmorrhagy had taken place, the animal was violently convulsed. This convulsion could only be *spinal*." (*Idem*, p. 44.)

Convulsion arising from cerebral disease, although often connected with insensibility, denotes an affection of a different part of the nervous system, and is indeed in general to be ascribed to what Dr. Hall calls "counter pressure." Fodera produced it in animals by pressing downwards *through* the brain on the medulla oblongata, but not by pressing laterally *on* the brain, even when he crushed it completely. In "a little girl aged thirteen months, the croup-like convulsion returned repeatedly, until one day, when the bones of the cranium separated: the convulsion then ceased." Here, of course, the diseased action in the brain went on; but, when it ceased to exert pressure on the spinal cord, the spasm ceased to result from it. "In the case of an acephalous fœtus described by Mr. Lawrence, convulsion was produced by pressing on the medulla oblongata; and, in a case of meningitis given by Dr. Abercrombie, the anterior fontanelle became very prominent, and pressure on it produced convulsion. Hypertrophy of the brain also produces convulsion, except in the case in which

* We need hardly say that we regard the medulla oblongata as the highest portion of the spinal cord, not as a part of the brain.

the cranium grows with the encephalon;" in which case, of course, the "counter pressure" will be prevented. All this is exactly in accordance with experiments, particularly those of Dr. Wilson Philip.

There is another class of phenomena in which the spinal cord is essentially concerned, more habitually occurring in the sound state, and frequently perverted in disease, which demands notice here. These are the phenomena to which Dr. M. Hall has given the rather uncouth name of "excito-motory," and which he states as the effects of the "reflex function of the spinal cord;" a power neglected or misunderstood, as he thinks, by former physiologists. But there has been some misapprehension on this subject, which we shall endeavour to remove.

It cannot be necessary to remind our readers of the great class of movements in living animals,—so characteristic of animal life, as distinguished from vegetable,—so essential to many of the functions of life, to respiration, digestion, and all their subordinate phenomena,—and so often the subject of discussion among physiologists,—to which the name of Sympathetic Actions is usually given, because they are cases in which an impression is made on some part of the body, and the immediate result is, a muscular contraction, or series of such contractions, in a distant part, which is said to sympathize with that originally impressed. It is thus that the actions of the muscles of respiration are excited, either by changes in the lungs or by applications, such as ammonia, to the nostrils,—or by impressions, such as the dashing of cold water on the skin of the face or breast,—that the actions of coughing, sneezing, deglutition, vomiting, and the movements of the abdominal muscles, of the sphincter ani, of the levatores ani, and acceleratores urinæ, in expelling the contents of the rectum and bladder, are effected.

Dr. M. Hall cannot be ignorant of the statements and reasonings by which Dr. Whytt (supported in this argument by Haller and by Monro,) laboured to prove that these phenomena do not imply a necessary or uniform consent of parts; but that the parts on which, in these cases, the primary impressions are made are connected with those in which the resulting and useful contractions take place only in so far as certain *sensations* are excited through the former parts; which sensations, as he taught, are the immediate cause of the contractions in the latter parts. This doctrine has been substantially adopted by different subsequent authors, and among others by Sir Charles Bell, in relation to the respiratory movements; who has exposed himself, however, to some animadversion by further maintaining (what is no part of the doctrine, and is indeed clearly in opposition to the principles of Whytt,) that the reason of the sensations of the lungs and air-passages acting as peculiar stimuli of the muscles of respiration is, that they have a peculiar association, at their root, with the motor nerves, or certain of the motor nerves, of these muscles.

But, without entering at present on that part of Sir C. Bell's speculations, we have to observe, that when the discovery of Sir C. Bell, confirmed by Magendie and others, had established the distinction of sensitive and motor nerves, it followed, as a necessary consequence, if the doctrine of Whytt was admitted, that, in any sympathetic action, the sensitive nerves of the part which is primarily impressed, and the motor nerves of the part which is thrown into action, must be those concerned in producing the changes. And, when it was ascertained by Le Gallois, Magendie, Flourens, and others, that the parts of the nervous system essentially concerned in sensation do not extend higher than the medulla oblongata, it became obvious to every one who reflected on the subject at all that, if the doctrine of the dependence of sympathetic actions on sensation is true, it must be the spinal cord and medulla oblongata, not the brain, that must be concerned in producing that description of muscular actions.

Now, the phenomena to which Dr. M. Hall gives the name of Excito-motory are precisely those which come under the old denomination of Sympathetic; and which, we believe, have generally been thought to be dependent on sensation, either immediately or through the intervention of the simplest and most general *instinct* to which sensations naturally lead. When he says that "the incident excitor" (i.e. sensitive) "nerves, the medulla, and the reflex motor nerves, constitute the excito-motory system,"* he merely gives this name to what every disciple of Whytt and Haller must, since the discoveries above quoted, necessarily regard as the parts of the nervous system on which the sympathetic actions depend; and, as it is generally admitted that the needless multiplication of names is a great source of confusion and embarrassment in science, we feel ourselves constrained to protest against the adoption of this new phraseology.

It is true that Dr. Hall has stated facts in regard to the movements of this class which, in his opinion, show that sensation is not necessary to their production. But, if this were established, it would only prove that Whytt and others have been mistaken as to the conditions necessary for exciting the sympathetic actions; not that it is expedient to give them a new name.

We do not, however, consider it established that these phenomena are truly independent of sensation. Dr. Hall's reason for thinking so is simply this, that they may be seen, on certain irritations being applied to the skin or other membranes, after the brain has been destroyed or removed, and when, he thinks, "the sentient and voluntary functions are annihilated." Thus, when a

* Lectures, &c., p. 21.

horse was rendered insensible by a blow on the head with a poll-axe, he remained motionless, manifesting no evidence of sensation or volition, although pricked by a pin or a nail on any part of the surface of the skin; but his respiration, although at first suspended, was soon renewed and went on freely; when the eyelash was touched with a straw, the eyelids were forcibly closed; when the cornea was touched, the eyeball revolved outwards; and, when the verge of the anus was touched, the sphincter contracted forcibly, and the tail was raised. These phenomena all ceased when "the upper part of the medulla oblongata was destroyed by an instrument passed through the orifice made by the pollaxe." Here, according to our author, the blow of the pollaxe annihilated the sentient and voluntary functions; the excito-motory, or sympathetic, remained, but were instantly suspended by destroying the medulla oblongata. Again, in a frog, after the spinal cord is divided below the occiput, there is no trace of *spontaneous* motion, and, as Dr. Hall supposes, no sensibility; but, on pinching the toe with the forceps, both posterior extremities are moved; and, on pinching any part of the skin, there is a recurrence of what he calls the excito-motory phenomena. These are no longer excitable after the spinal cord is extracted or destroyed with a probe, although the muscles remain distinctly irritable, as is proved by the application of galvanism.

All this, as Dr. Hall observes, is wonderful; but we cannot help expressing our surprise at his adding, "and, I believe, hitherto quite unknown to physiologists." Such facts had been often observed and recorded, but were generally regarded as proofs that a certain degree of sensation *remains* in these circumstances; and we are by no means satisfied that that supposition is erroneous. Dr. Whytt states, e.g., "A frog lives and moves its members for half an hour after its head is cut off; nay, when the body of a frog is divided in two, both the anterior and posterior extremities preserve life and a power of motion for a considerable time." (*Essay on the Vital and Involuntary Motions, &c. Edinb.* 1751, p. 384.) Again, "if one of the muscles of the leg of a frog is irritated after cutting off its head, almost all the muscles of the legs and thighs are brought into action, *if the spinal marrow be entire;* but, as soon as it is destroyed, although the fibres of such muscles as are themselves irritated are affected with a weak tremulous motion, yet the neighbouring muscles remain at perfect rest." (*Observations on Irritability in Nerves*, p. 291.) He supposed that in such cases sensation remains. "We have no other way," says he, "to satisfy ourselves that an animal is alive or endued with feeling, than by observing whether it shews an uneasiness when anything hurts or tends to destroy any of its parts, and an endeavour to remove or avoid it. Since, therefore, the bodies of vipers make just the same kind of motions, when pricked with a sharp instrument, two or three days after losing their head, heart, and other bowels, as if they were entire, we are naturally led to conclude that they are still in some sense alive and *endued with feeling*, i. e. animated by a *sentient principle*." (P. 388.) Haller mentions similar facts, even in warm-blooded animals, and draws the same inference. Mr. Mayo is more cautious as to the inference that sensation in these circumstances remains; but was fully aware, long before Dr. Hall wrote, of the necessity of the spinal cord in such motions.

"When the skin of the lower extremities of an animal," says he, "is irritated after the division of the spinal cord in the back, muscular action occurs, limited to the muscles of the inferior extremities. I have varied this experiment by dividing the spinal cord at once in the neck and back, upon which these unconnected nervous centres exist, and the division of the skin in either part produces a convulsive action of the muscles of that part alone. It may be said that this experiment of making different nervous centres by division of the spinal marrow, admits of explanation on supposing the principle of volition, and sensation also, to continue for a short period extended to the portions separated from the brain; a conjecture consistent with, though not established by, the very curious fact that the movement of the leg of an animal thus circumstanced, when the sole of its foot is irritated, is accurately the gesture which the animal employs when, in undisputed possession of sensation, it retracts its limb from a similar aggression." (*Anatomical and Physical Commentaries*, No. 2, p. 18.)

Le Gallois, after describing similar experiments which he had very frequently made on young rabbits, says, "La section de la moëlle a evidement etabli, dans le même animal, deux centres de *sensations* bien distincts et independans l'un de l'autre; l'on pourroit même dire deux centres de volonté, si les mouvemens que fait le train de derrière, *quand on pince la queue*, ou bien une des pattes posterieures, supposent la volonté de se soustraire au corps qui le blesse." (*Experimens sur la Principe de la Vie*, p. 60.) It is well known that Le Gallois proved by numerous experiments that these indications, as he thought them, of sensation and of intuitive or voluntary motion in the trunk and limbs, remaining after the head of an animal has been cut off, or its spinal cord *divided*, are uniformly and instantaneously destroyed by *crushing* the spinal cord. (See *Experimens, &c.*, p. 32 et seq.) A Dutch author, Van Deen, treats the subject fully, and explains distinctly the difference between these movements and the simple effects of irritability. "Stimulus ranæ decapitatæ applicatus stimulat *nervos sensorios*, qui hunc stimulum acceptum in centrum (*medullam spinalem*) propagantes, hic solito munere funguntur, et reactionem, scilicet motûs stimulum procreant." (*De differentia et nexu inter Nervos Vitæ Animalis et Vitæ Organicæ*, p. 76.) And, not to overload the subject with quotations, we may merely add that these indications of what Dr. M. Hall calls the excito-motory power, or the reflex function of the spinal cord, and which he thinks independent of sensation, are the very same phenomena which Cuvier took for his test of the *continuance of sensation,* in reporting on the series of experiments undertaken by Flourens to ascertain how much of the cerebro-spinal axis, beginning from its anterior extremity, might be destroyed without necessarily interrupting the sensations of any part of the body. (See *Rapport sur le Memoire de Flourens, &c.* in the *Recherches Experimentales* of this author, p. 77-79.)

Dr. Hall speaks in some places of the "excito-motory system of nerves" as if they were a separate set of nerves, distinct from those of sensation, and of which the respiratory nerves are a part; and, in the experiment mentioned on the horse, it would seem that the eyelids and the verge of the anus were the only parts of the surface of the animal which exhibited the phenomena in question, and appeared therefore, according to this language, to possess nerves of the excito-motory system. But this distinction cannot be observed; for, in his own experiments on the frog and turtle, (p. 26,) in his reference to the effect of cold water dashed on the surface, (ibid.,) and in many of the experiments of other authors already quoted, it distinctly appears that phenomena of this description may be excited, even in warm-blooded animals, through any of the nerves of common sensation on the surface of the body; so that the difference between the eyelids and verge of the anus and other parts of the surface in this respect must be one of degree only, not of kind; and, if we make a system of excito-motory nerves, we must include in that system all the nerves of common sensation, and probably all those of voluntary motion.

The only truly original observations on this subject which we have noticed in the work of Dr. Hall are these: 1. That "the whole tone of the muscular system is the result of an excito-motory function;" i. e. is dependent on an agency of the spinal cord, probably excited by sensitive nerves. If we remove the tail and rectum from a recently decapitated turtle, the sphincter retains its circular form, the tail its firmness; phenomena which cease entirely on withdrawing the portion of spinal marrow remaining within the caudal spinal canal." 2. That the effect of "gently withdrawing" the spinal cord upon this description of muscular actions is as great as that of violently crushing it." (*Lect.* i.)

But, although we deny *in toto* the originality of Dr. Hall in observing these vital actions, and very much doubt the propriety of giving them a new name, we do not mean to detract from his merit in fixing the attention of physiologists upon them, and on the agency of the spinal cord in producing them; and likewise in casting doubts on the doctrine of their dependence on sensation. Whether they really indicate sensation or not, is a point which can only be decided by observations in cases of injury or disease of the human body; and we observe two statements in the works before us which we admit to be in favour of the supposition that sensation does not necessarily intervene between the impression on the one set of nerves and the muscular action excited, so long as the spinal cord is entire, through the other. The first is the observation by Dr. Ley of a person in whom the par vagum appeared to be diseased; the lungs suffered in the usual way, in consequence, and the patient had "evidently laborious breathing," but said distinctly that he felt no uneasiness in the chest. (P. 417.) The second is a case given by Mr. Mayo, of palsy and complete loss of sensibility in one leg, in which, nevertheless, irritating the toes caused retraction of the leg, without, as he was assured, any consciousness on the part of the patient. (*Outlines*, p. 154.) For this we have looked in vain in several similar cases, although we have seen more than once the curious phenomenon of muscles acting in obedience to sensations, (particularly in yawning;) i. e. in obedience to the spinal cord, when they were palsied to voluntary efforts, i. e. to changes originating in the brain.

We do not regard these cases as decisive of the point in question, but we must admit that, as it is only by exciting a peculiar unknown change in the spinal cord that impressions on any sensitive nerves excite sensations in the mind, so it is quite possible that these changes in the spinal cord may not be wholly prevented from taking place in cases where disease of the brain, or injury of the cord itself, so far affects the spinal cord as to prevent sensations from being felt; and that these changes, not the sensations which in the natural state accompany them, may be the causes of the sympathetic, or excito-motory, actions. Still the important fact regarding these actions, which we conceive to be established by Whytt and his followers is that, if not, strictly speaking, dependent on sensations, they are dependent on changes in the spinal cord which, in the natural state, attend and indicate sensations, and which are useful in the animal economy only as conducing to the relief or removal of the sensations which they denote.

We regard the excito-motory phenomena of Dr. M. Hall, therefore, as only further indications of the powers which are given to the spinal cord to enable it, in the living body, to minister to the functions of sensation and of instinctive and voluntary action. Dr. M. Hall states, that "the whole order of spasmodic and convulsive diseases belongs to the excito-motory division of the nervous system;" which, considering that this system must necessarily include the spinal cord and all the nerves of common sensation and voluntary action, can hardly be denied. He then goes on to explain that such diseases are sometimes excited by causes acting in the central masses of the nervous system, when he calls them *centric;* sometimes by causes acting on the excitor, or sensitive nerves, at a distance from the larger masses, when he calls them *eccentric;* and in a few cases by irritation of the motor nerves themselves: all which is very true; but we do not see that it differs materially from the common statement, that spasms may be excited directly by injury or disease of the motor nerves, or of the motor portion of the spinal cord; less directly, and not necessarily or uniformly, by injuries of the brain; and sympathetically, in certain cases, by impressions made in the course, or still more at the extremities, of sentient nerves, and which act *sympathetically* on certain muscles. An important observation, which he has not illustrated at such length as its practical usefulness would have justified, but which did not escape the notice of Dr. Whytt, is that when spasmodic affections in disease are excited in this last way, their nature and seat are very often determined by the sympathetic actions resulting in health from excitement of the same sensitive nerves: in fact, as we believe, by the nature and the natural effects of the *sensations* which these nerves are fitted to transmit. Thus, we have sneezing from irritation of certain branches of the 5th nerve, although there be nothing to expel from the nose; coughing from irritation of certain branches of the 8th, although there be nothing to expel from the air-passages; tenesmus or strangury from irritation of the sensitive nerves of the great intestines or of the bladder, although there be nothing to expel from these viscera; and vomiting from irritation of the brain, fauces, heart, liver, intestines, kidneys, uterus, or even surface of the body, although there be nothing to expel from the stomach; because, in all these cases, the sensations which naturally and usefully prompt to these sympathetic actions are excited in an unwonted manner by these different irritations. The practical use of this piece of pathology in guiding us to the seat of disease, and to the use, or perhaps more frequently to the disuse, of remedies, is sufficiently obvious. And, in all these cases, it is now certain that the spinal cord and medulla oblongata, not the brain or cerebellum, are the media of transmission of the stimuli which ultimately excite the muscles. If Dr. Hall is desirous of applying the term Reflex Function of that organ to these cases, we see no objection to his doing so; but we cannot admit, either that we owe to him our knowledge of the principle, or that it is best expressed by ascribing it to the agency of the "excito-motory division of the nervous system."

We must reserve for a future Number the observations suggested to us by the facts, in the works before us, which belong to the last division of our subject, the Pathology of the Brain and Cerebellum.

